# Morocco and the Canary Islands as Health or Holiday Resorts

**Published:** 1906-08-25

**Authors:** 


### MOROCCO AND THE CANARY ISLANDS AS HEALTH OR HOLIDAY RESORTS.

To the seekers after health or rest sea-trips are becoming increasingly popular. We were lately induced to try the effect of a trip to the coast of Morocco and the Canary Islands in one of Forwood Brothers' steamers. These vessels sail from the Morocco Wharf, Wapping, and our first introduction to the said wharf was a demand for 5s. for being allowed to cross a few yards of platform with our luggage, which consisted of two small trunks and three handbags. There would seem to be no appeal from this absurd charge, and we think that Messrs. Forwood Brothers should either pay these charges or remove their place of departure elsewhere. Fancy a railway company charging their passengers 1s. a package for being allowed to cross their platforms, or fancy any charge being made at a Liverpool or Southampton landing-stage!

From London these steamers go to Dartmouth, to take in coal for the voyage out and home; and then proceed to Gibraltar or to one of the Morocco ports, which they reach in about seven days, and afterwards steam along the Morocco coast, calling at several ports, until they reach Mogador. The time allowed on shore is usually only a few hours; but it is generally long enough to see the towns, and in the present state of affairs in Morocco the visitor would be well advised not to stray far outside of the towns, in which, or at least in some of which, he will find sufficient to interest him. At any rate, he will meet with new experiences. He will find the natives moving about in their long flowing robes; he will contrast the narrow, dirty, dusty streets with the dazzling whiteness of the towns as seen from the steamer's deck; and he will find bits of old Portuguese architecture close to the Moorish; he will be pestered with guides and with dealers in "curiosities," some of the things offered for sale being veritable Moorish and others evidently "Brummagem." The traveller may also be interested in the deck passengers, who are carried from one Morocco port to another, and for whom an awning is rigged over one of the hatches. In these Morocco ports the harbours are of such primitive construction that it is not always quite safe to risk the passage through the surf. Sometimes it is impossible to land; and although the shore boats are large and are well manned by the Moors, they may be over-crowded by passengers, and it would be infinitely preferable if Messrs. Forwood Brothers would provide an electric launch of their own. Unless it be at Tangier, few English invalids would dream of making any town in Morocco their winter residence. True, there is a French hotel in Mogador, and another one a few miles along the coast, to which time did not permit a visit, but at which, we believe, accommodation could be obtained; and we need hardly say that the climate of Mogador is as good, or perhaps better, than that of most of the more famous winter resorts. It may become popular when a reliable police force is in existence, when superior accommodation is provided, and when the harbour is improved. A large island lies in front of Mogador, and Nature seems to have intended it for a harbour, while Art has so far given but little aid.

From Mogador the steamer strikes straight out for the Canary Isles. Teneriffe may be the first place touched at, and the landing will be at Santa Cruz, a town of more than 20,000 inhabitants and the capital of the Canaries. It is characteristically Spanish in appearance. Many of the houses have "patios" or central courts, and "miradores" on the roofs are quite common. The hotel accommodation at Santa Cruz is quite good. Among others are the Quisisana, on the brow of a hill overlooking the town and the sea, from 14s. a day; the Comacho, in the town, from 9s.; the Victoria English Hotel, much cheaper. Other hotels, 5s. or 6s., at Laguna, a town of 12,000 inhabitants, about five miles from Santa Cruz; and the Tacoronte Hotel, beautifully situated, about twelve miles from Santa Cruz. Some of the hill hotels are managed in connection with hotels in Santa Cruz. Tacoronte is about half-way to Oratava, in which many English people live during the winter, and in which the hotel accommodation is said to be good; but here again time prevented a visit.

Grand Canary lies about fifty miles west by south of Teneriffe. Las Palmas is the chief town, and its port is Puerto de la Luz. This is a little over three miles from the town. Plenty of cabs are to be had, and a light railway runs every hour or so. Between the port and the town are the Metropole Hotel and the Santa Catalina Hotel, the former being on the seashore and the latter a little way inland on the opposite side of the road. In the town is Quiney's English Hotel, from 6s. a day. This is in connection with the Bella Vista Hotel, six miles from Las Palmas, and is built on the hillside about 1,200 feet above sea-level. As regards appearance, Canta Cruz is a finer town than Las Palmas, but the climatic conditions of the two islands do not differ much. The rainfall at Las Palmas shows a somewhat lower average than at Santa Cruz; but even at the latter the mean average for January is under 2 inches, and it is less during February and during March, and the number of days during which rain fell in these months is respectively eleven, nine, and eight. The mean daily temperature at Santa Cruz is also slightly higher than in Grand Canary, and for January it is 74°, for February 63°, and for March 64°. There are occasional dust or sand storms. Many excursions by carriage or tram may be made in the Canary Islands, and a round of the isles may be taken on the interinsular steamers at the charge of £3 10s. It is possible to break the voyage by the Forwood steamers, and remain one week or any number of weeks up to twenty-four in any of the islands, but an extra charge is made for this privilege.

Madeira is a day's sail from Grand Canary. The steamer drops anchor opposite Funchal, and the traveller cannot fail to be charmed with his first view of this lovely little town, a charm which is not lessened, but rather increased, when he steps on shore. The town is unlike all others of its size we have visited. There are delightful open spaces and gardens, and the profusion of flowers and sub-tropical vegetation are most pleasing to the eye of the invalid who has left England in winter or early spring. There is, too, a singular and blissful quietude in the town—a quietude that reminds one of Venice. The "carro," which we may call the gondola of Funchal, glides on its runners as evenly over the small smooth pebbles with which the streets are paved as does the gondola over the canals and lagoons of the city on the Adriatic. The "carro" is drawn by a pair of bullocks, and the driver guides the animals with a long goad, much as the gondolier does with his long oar, except that one applies his steering gear to the head of the bullocks and the other to the stern of the boat.

In Funchal there are plenty of good hotels, the charges in which range from 7s. a day up to £1. The climate is wonderfully equable. Omitting fractions, the average daily temperature for January, February, and March is 60°, and the mean daily range is under 10°. The rainfall is considerably higher than in the Canaries, and the average number of days on which rain fell was in January 12, in February 6, and in March 8. Fogs, or mists, or dust storms are practically unknown.

Invalids who intend visiting any of these islands will like to know that during the season there are three English medical men in Las Palmas, two in Oratava, one in Santa Cruz, and six in Funchal.

From Madeira to London by the Forwood line steamers takes about seven days. The round trip, without change of steamers, takes about twenty-five days, and the fare is £23 2s., or if a deck cabin be taken, £25 4s. Berths in inside cabins may be had for £21; but as this article is intended as a guide for those who take voyages for health's sake, these "inside" cabins are out of the question. The trip cannot be said to be cheap; in fact, it is dear compared to other cargo lines. For instance, the Booth trip to Portugal of a similar length is much better done for less money. Furthermore, to the Forwood Brothers' trip £3 2s. for excursions and the wine bill must be added, whereas the excursions and landings and table wine are included by the Booth line. Again, the Papayamin lines' trip to the Mediterranean every fortnight costs £24 for about forty-five days. The General Steam Navigation Company's trip to the Mediterranean is £20 for six weeks; Elder Dempster's trip to the Canary Isles, including fourteen days' hotel expenses, is £15; and the Moss line offer eight weeks or more for £35. During the summer months cheap trips are run from Southampton by the Union-Castle line.

To the average traveller the stated tonnage of a vessel conveys no clear idea of its size, and it would be decidedly useful if the length and breadth of steamers were given in advertisements. Certainly the Board of Trade should insist that all vessels taking passengers should be compelled to advertise their registered tonnage, even if other tonnages are given. For instance, the newest steamer of the Forwood line is stated in the Board of Trade certificate to be 1,022 tons register, in Dowling's *Ships and Shipping* she is given at 1,618 tons, and in Forwoods' own handbook at 2,800. The owners would be well advised to attend to this matter, and also to alter the day of arrival in London from Sunday to a weekday. A Bradshaw's Railway Guide might be kept on all these steamers.

